# MASLD in the oldest-old: lack of association with cognition or functional autonomy and poor predictive utility of the hepatic steatosis index

**DOI:** 10.1007/s11357-025-02060-3

**Published:** 2025-12-24

**Authors:** Maximilian Joseph Brol, Martin Groth, Frank Erhard Uschner, Juliana Stadtmann, Tina Schomacher, Jonel Trebicka, Michael Praktiknjo, Elena Vorona

**Affiliations:** 1 Department of Internal Medicine B, University Hospital Muenster, Albert-Schweitzer-Campus 1, A148149 Muenster, Germany; 2 Department of Internal Medicine IV, Mathias Spital Rheine, Rheine, Germany; 3 European Foundation for the Study of Chronic Liver Failure-EF Clif, Barcelona, Spain

**Keywords:** MASLD, Sarcopenia, Steatosis, Elderly, Older adults, Functional assessment

## Abstract

Metabolic dysfunction-associated steatotic liver disease (MASLD) is a prevalent condition increasingly recognized in aging populations. However, its clinical relevance in older adults—particularly in relation to sarcopenia, cognitive function, and rehabilitation outcomes—remains poorly defined. This retrospective observational study included in-patients admitted for geriatric rehabilitation in 2022 with available abdominal ultrasound. MASLD was defined according to AASLD/EASL criteria. Demographics, comorbidities, medications, and the hepatic steatosis index were recorded. Cognitive function (MMSE, clock-drawing, GDS), hand grip strength, mobility (Tinetti, Timed Up and Go), and functional status (Barthel Index, discharge autonomy) were assessed. Analyses included regression and AUROC, with significance at p below 0.05. MASLD was present in 49.6% of the study population. No significant association was observed between MASLD and cognitive impairment. Patients with higher hand grip strengths showed higher proportions of MASLD (p = 0.01). In this cohort, hepatic steatosis index has poor capacity to detect hepatic steatosis (AUC = 0.503, 95%-CI: 0.437–0.570). Furthermore, MASLD did not predict poorer functional outcomes following the rehabilitation stay. In adjusted models, Barthel Index —but neither MASLD nor hand grip strength— was independently associated with reduced functional autonomy at discharge (p = 0.006). In elderly patients, MASLD was associated with diabetes mellitus and higher hand grip strength, while sarcopenia was linked to a lower prevalence of steatosis. MASLD showed no association with cognitive performance, functional outcomes, or prediction by the hepatic steatosis index. These findings highlight age-specific characteristics of MASLD requiring tailored clinical approaches.

**Graphical Abstract:**

**Figure:**
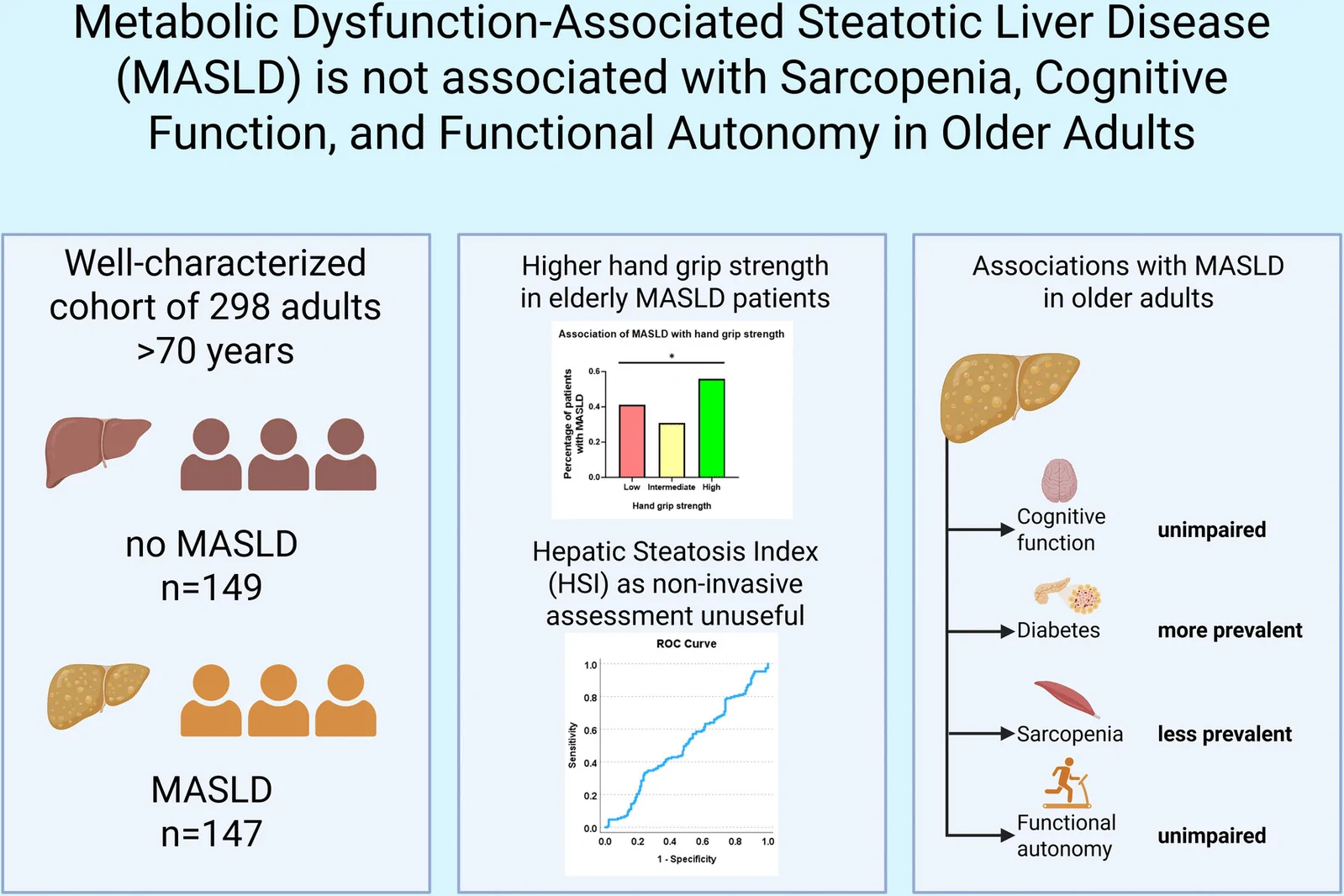

**Supplementary Information:**

The online version contains supplementary material available at https://doi.org/10.1007/s11357-025-02060-3.

## Introduction

Metabolic dysfunction-associated steatotic liver disease (MASLD) has emerged as the most prevalent chronic liver condition globally, with rising incidence not only in middle-aged populations but also among older adults. MASLD encompasses a spectrum ranging from simple steatosis to metabolic dysfunction-associated steatohepatitis (MASH), fibrosis, and cirrhosis, and is increasingly recognized as a multisystem disease with metabolic, cardiovascular, and renal consequences [1]. Although substantial attention has been paid to these systemic complications, the implications of MASLD in the context of aging remain underexplored [2].

Sarcopenia, characterized by the age-related loss of muscle mass and function, poses a significant threat to independence and quality of life in the elderly [3]. Emerging evidence suggests that MASLD and sarcopenia may share common pathophysiological pathways, including chronic inflammation, insulin resistance, and mitochondrial dysfunction [4, 5]. Cross-sectional studies in younger populations have reported associations between MASLD and reduced muscle strength [4, 6, 7]. Yet, data in older cohorts are limited.

Similarly, cognitive impairment is a major concern in geriatric medicine, ranging from mild cognitive decline to dementia. MASLD has been implicated preclinically in neurocognitive dysfunction through mechanisms such as systemic inflammation, endothelial dysfunction, and altered lipid metabolism [8]. However, the extent to which MASLD contributes to cognitive deficits in older individuals remains unclear.

Given the aging global population and the increasing burden of MASLD, understanding its associations with geriatric syndromes is of growing importance. Clarifying these relationships could offer valuable insights into the management and prognosis of older patients with MASLD. To address this knowledge gap, we formulated the following hypotheses: (i) MASLD in older adults is associated with reduced hand grip strength, serving as a surrogate marker for sarcopenia; (ii) MASLD in older adults is associated with cognitive impairment; (iii) MASLD in older adults is associated with poorer functional outcomes following an inpatient geriatric rehabilitation stay.

## Methods

### Study design and population

**Fig. 1:**
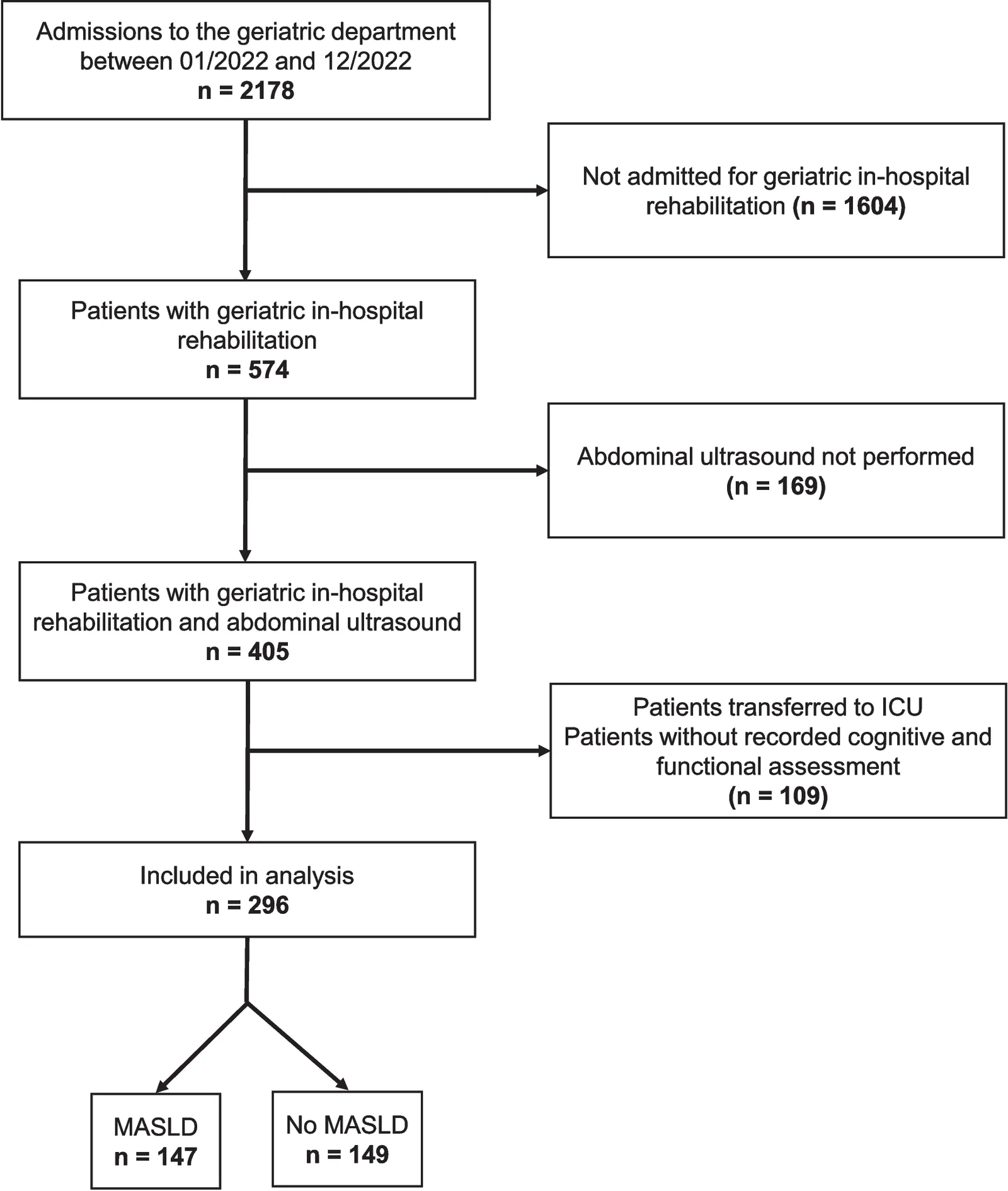
Study flowchart**.** Study flow diagram illustrating screening and allocation of the study cohort. A total of 2178 admitted patients were screened. Patients were excluded if they were not admitted for geriatric in-patient rehabilitation (1604 patients) or if an abdominal ultrasound was not available (further 196 patients) or if the patients were transferred to ICU during the stay or had not undergone cognitive and functional assessment at admission (109 patients). In total, 296 patients were included, of which 147 had MASLD

This study employed a retrospective observational design. All patients admitted to the Department of Geriatrics at Department of Internal Medicine IV, Mathias Spital Rheine, Rheine, Germany in the year 2022 were screened for eligibility. Patients were included if an abdominal ultrasound report documenting the presence or absence of hepatic steatosis was available within the observational period. MASLD was diagnosed according to the current AASLD and EASL recommendation [9]. MASLD was diagnosed only if both of the following criteria are met: (1) evidence of hepatic steatosis on abdominal ultrasound and (2) the presence of at least one of the following cardiometabolic risk factors (Overweight or obesity (BMI ≥ 25 kg/m^2^) in non-Asians; Type 2 diabetes mellitus or prediabetes or treatment with antidiabetics; arterial hypertension or a blood pressure > 130/85 mmHg or treatment with antihypertensives; dyslipidemia (elevated triglycerides (> 1.7 mM) and/or reduced HDL cholesterol < 1 mM or lipid-lowering treatment). Patients without geriatric rehabilitation or an ICU stay were excluded from the study (Fig. 1).

### Data collection

Medical records were reviewed to extract demographic data, clinical diagnoses (based on ICD codes), and information on concomitant medications at the time of admission. A total of 21 drug or drug classes were recorded: ACE inhibitors, AT1 receptor antagonists, betablockers, calcium antagonists, high-ceiling diuretics, aldosterone antagonists, thiazide diuretics, statins, antidiabetics, extended-release insulin, antiplatelet therapy, oral anticoagulants, heparins, selective serotonin reuptake inhibitor (SSRI), proton pump inhibitors (PPI), vitamin D, steroids, L-Thyroxine, metamizole as well as class II and III opioids. All comorbidities, including sarcopenia, were identified based on ICD codes documented in the patients’ medical records..

### Hepatic steatosis index

To evaluate whether currently used non-invasive assessments can detect hepatic steatosis without ultrasound, the hepatic steatosis index (HSI) was calculated for each patient. The HSI utilizes routinely available clinical and laboratory parameters, with established cutoff values of < 30 indicating a low probability and > 36 indicating a high probability of MASLD. The index is calculated based on a formula incorporating alanine aminotransferase (ALT), aspartate aminotransferase (AST), body mass index (BMI), sex, and the presence of diabetes mellitus [10]. It is calculated using the following formula:

$$HSI=8x \left(\frac{AST\left[\frac{U}{l}\right]}{ALT \left[\frac{U}{l}\right]}\right)+BMI \left[\frac{kg}{{m}^{2}}\right]+2 \left(if\, female\right)+2 (if\, diabetes)$$

### Cognitive assessment

Cognitive function was evaluated using standard-of-care procedures by the following standardized tools:

Mini-Mental State Examination (MMSE): The MMSE is a 30-point questionnaire assessing orientation, registration, attention and calculation, recall, and language. Scores range from 0 to 30, with lower scores indicating greater cognitive impairment [11].

Shulman Clock Drawing Test: Patients were asked to draw a clock showing a specific time. The drawing was scored on a 6-point scale, with lower scores indicating greater impairment in visuospatial and executive functioning [12].

Geriatric Depression Scale—Short Form (GDS-15): The GDS is a 15-item self-report screening tool used to assess depressive symptoms in older adults. Scores ≥ 5 suggest possible depression, while scores ≥ 10 are considered indicative of clinically relevant depressive symptoms [13].

### Sarcopenia assessment

Hand Grip Strength: Muscle strength was assessed using a hand dynamometer (measured in kilograms). The highest value from three trials per hand was recorded, with the best overall score used for analysis.

Tinetti Performance-Oriented Mobility Assessment (POMA): This tool evaluates balance and gait. The maximum score is 28 points, with lower scores indicating increased risk of falls and impaired mobility [14].

Timed Up and Go (TUG) Test: Patients were instructed to rise from a chair, walk three meters, turn around, return, and sit down again. The total time taken (in seconds) was recorded. Longer times indicate decreased mobility and higher fall risk [15].

### Functional assessment

Functional independence was evaluated both prior to admission and at discharge:

Barthel Index: This index measures performance in 10 activities of daily living (e.g., bathing, dressing, toileting, mobility). Scores range from 0 to 100, with higher scores reflecting greater independence.

Autonomy at discharge was assessed using an ordinal scale with three categories: autonomous, semi-autonomous, and dependent. Patients classified as autonomous were those who returned to their own home, either living alone or with a spouse, without receiving support from an outpatient nursing care service. Semi-autonomous patients were discharged either to their private residence with the assistance of outpatient nursing care, to a senior shared apartment, or to an assisted living facility that provided nursing support. Patients deemed dependent were discharged to a nursing home, short-term or long-term care facility, or a rehabilitation center, or had deceased prior to discharge.

### Ethical approval

This study was conducted in accordance with the Declaration of Helsinki and approved by the ethics committee of the Medical Board Westfalen-Lippe. The requirement for patient consent was waived, due to the retrospective nature of the study. Ethical approval was granted under file reference 2023–525-f-S. Analyses were carried out on anonymized data.

### Statistical analysis

All statistical analyses were performed using IBM SPSS Statistic, Version 29.0 (IBM, Armonk, NY, United States) or GraphPad Prism, Version 10.0 (GraphPad Software Inc, La Jolla, CA, United States). Descriptive statistics were reported as medians with interquartile ranges or absolute/relative frequencies, as appropriate. Group comparisons were conducted using chi-square tests for categorical variables and non-parametric Mann–Whitney U tests for continuous variables.

Associations between variables and hepatic steatosis were assessed using univariable and multivariable binary logistic regression models. Discriminative ability was evaluated using area under the receiver operating characteristic curve (AUROC) analyses. Accuracy was calculated with the following formular: Accuracy = (Sensitivity * Prevalence) + (Specificity * (1—Prevalence)). For comparisons involving more than two groups, the Kruskal–Wallis test was applied. For associations of variables with ordinally scaled variables, ordinal regression analysis was performed. A p-value < 0.05 was considered statistically significant.

## Results

### General characteristics of the study cohort

A total of 296 elderly patients were included in the analysis, with 149 patients in the non-steatosis group and 147 in the steatosis group. Patients’ characteristics are displayed in Table 1. The median age was comparable between groups (85 years [81–89] vs. 85 years [81–87], *p* = 0.407). Sex distribution was also similar, with females comprising 65.8% of the non-steatosis group and 66.7% of the steatosis group (*p* = 0.871).

**Table 1:** Patients’ characteristics

	No MASLD (*n*=149) N(%), median (IQR)	MASLD (*n*=147) N(%), median (IQR)	*p*-value
Age	85 (81-89)	85 (81-87)	0.407
Sex (female)	98 (65.8%)	98 (66.7 %)	0.871
BMI	26.4 (23.6- 29.0)	27.9 (24.4-30.9)	0.022*
Creatinine	1.05 (0.78-1.55)	1.05 (0.80-1.65)	0.657
ALT	15 (11-22)	17 (12-26)	0.050
AST	22 (18-31)	23 (18-34)	0.587
GGT	38 (19-96)	48 (23-100)	0.062
AP	99 (79-135)	95 (74-124)	0.385
Bilirubin	0.48 (0.33-0.77)	0.45 (0.32-0.64)	0.249
INR	1.21 (1.04-1.49)	1.13 (1.04-1.39)	0.208
Triglycerides	94 (72-135)	132 (95-169)	0.002*
Total cholesterol	146 (119-172)	161 (129-188)	0.278
Total protein	6.88 (6.39-7.43)	6.73 (6.27-7.29)	0.168
Albumin	3.72 (3.26-4.01)	3.69 (3.35-4.04)	0.832
Hemoglobin	11.9 (10.4-13.3)	11.8 (9.5-13.3)	0.452
Ferritin	155 (70-331)	133 (59-343)	0.239
Vitamin D	14.0 (8.0-25.7)	13.8 (8.9-30.5)	0.332

Data are presented as number and frequencies or median and interquartile range. P<0.05 were considered significant (*). Chi-Square test for categorial variables and Mann-Whitney U test for scaled variables were used

Body mass index (BMI) was significantly higher in patients with steatosis (median 27.9 [24.4–30.9] vs. 26.4 [23.6–29.0], *p* = 0.022), suggesting a potential association between increased BMI and hepatic steatosis. Serum triglyceride levels were also significantly elevated in the steatosis group (132 [95–169] vs. 94 [72–135], *p* = 0.002).

Liver function tests showed slightly higher values in the steatosis group, with alanine aminotransferase (ALT) approaching statistical significance (*p* = 0.050), and gamma-glutamyl transferase (GGT) showing a similar trend (*p* = 0.062), while aspartate aminotransferase (AST), alkaline phosphatase (AP), bilirubin, and international normalized ratio (INR), did not differ significantly between groups.

No significant differences were observed in renal function (creatinine), hematologic parameters (hemoglobin, ferritin), or nutritional markers (total protein, albumin, vitamin D) between the two groups.

### Sarcopenia is negatively associated with hepatic steatosis in elderly patients

Comorbidities were compared between patients with and without hepatic steatosis in Table 2. Among the evaluated conditions, diabetes mellitus and sarcopenia were significantly altered between patients with and without hepatic steatosis.

**Table 2:** Comorbidities

	No MASLD (*n*=149) N(%)	MASLD (*n*=147) N(%)	*p*-value
Sarcopenia	45 (30.2%)	29 (19.7%)	0.037*
Diabetes	43 (28.9%)	59 (40.1%)	0.041*
Osteoporosis	24 (16.1%)	17 (11.6%)	0.258
Coronary artery disease	41 (27.5%)	43 (29.3%)	0.741
Gout	2 (1.3%)	2 (1.4%)	0.989
Chronic pain syndrome	56 (37.6%)	46 (31.3%)	0.571
Anemia	67 (64.4%)	66 (69.5%)	0.450
Depression	24 (16.1%)	24 (16.3%)	0.959
Sleep apnea disorder	14 (9.4%)	17 (11.6%)	0.542
Atrial fibrillation	69 (46.3%)	57 (38.8%)	0.190
Chronic heart failure	77 (47.7%)	82 (55.8%)	0.479
Chronic kidney disease	78 (52.3%)	66 (44.9%)	0.200
Parkinson’s disease	6 (4%)	9 (6.1%)	0.411
Chronic obstructive pulmonary disease	27 (18.1%)	27 (18.4%)	0.956
History of myocardial infarction	13 (8.7%)	13 (8.8%)	0.971
Arterial hypertension	117 (78.5%)	125 (85%)	0.147
Cerebrovascular disease	35 (23.5%)	31 (21.1%)	0.620
Dementia	52 (34.9%)	47 (32.0%)	0.594

Data are presented as number and frequencies. P<0.05 were considered significant (*). Chi-Square test for categorial variables was used

Specifically, diabetes was present in 40.1% of patients with steatosis compared to 28.9% without steatosis (p = 0.041). Conversely and surprisingly, sarcopenia was more common in the non-steatosis group (30.2%) than in those with steatosis (19.7%), also reaching statistical significance (p = 0.037). Regarding other comorbidities, no significant differences were found between the groups. These results highlight a potential link between hepatic steatosis and certain metabolic or musculoskeletal comorbidities in this elderly cohort.

### L-thyroxine and class III opioids are associated with the presence of hepatic steatosis in elderly patients

In order to evaluate the association of concomitant medication and the presence of hepatic steatosis, univariable and multivariable binary logistic regression analyses were performed.

In the univariable analysis, shown in Table 3, none of the drug categories showed statistically significant associations with hepatic steatosis except for class III opioids, which were associated with a significantly reduced odds of steatosis (OR = 0.529, 95% CI: 0.314–0.892; p = 0.017) and l-thyroxine, which was associated with a significantly increased odds of steatosis. Extended-release insulin showed a trend toward increased odds of steatosis (OR = 3.128, 95% CI: 0.621–15.755), although this did not reach statistical significance (p = 0.167). In the multivariable model, two medications remained significantly associated with hepatic steatosis. L-thyroxine use was associated with increased odds (OR = 1.853, 95% CI: 1.058–3.247; p = 0.031), while class III opioids continued to demonstrate a protective association (OR = 0.505, 95% CI: 0.297–0.857; p = 0.011).

**Table 3:** Uni‐ and multivariable binary logistic regression analysis of concomitant medication associated with MASLD

	Univariable binary logistic regression				multivariable binary logistic regression			
Drug	P	Odd`s ratio	Confidence interval		P	Odd`s ratio	Confidence interval	
ACE inhibitors	0.742	1.083	0.672	1.746				
AT1 receptor antagonists	0.255	1.360	0.801	2.307				
No betablockers	0.415	1.220	1.969	0.756				
Calcium antagonists	0.340	1.271	0.777	2.078				
High-ceiling diuretics	0.386	1.232	0.769	1.972				
No aldosterone antagonists	0.662	1.119	1.848	0.677				
thiazide diuretics	0.312	1.493	0.686	3.247				
No statins	0.469	1.186	1.883	0.747				
antidiabetics	0.224	1.362	0.827	2.244				
Extended-release insulin	0.167	3.128	0.621	15.755				
No antiplatelet therapy	0.953	1.015	1.692	0.609				
No oral anticoagulants	0.412	1.211	1.912	0.767				
heparins	0.974	1.015	0.426	2.419				
SSRI	0.509	1.321	0.579	3.013				
PPI	0.946	1.019	0.600	1.728				
Vitamin D	0.803	1.060	0.670	1.679				
Steroids	0.685	1.160	0.567	2.373				
*L_Thyroxine*	*0.047*	*1.748*	*1.007*	*3.033*	0.031	1.853	1.058	3.247
No metamizol	0.120	1.445	2.304	0.908				
Class II opioids	0.568	1.372	0.464	4.055				
*No class III opioids*	*0.017*	*1.890*	*3.185*	*1.121*	**0.011**	**1.980**	**3.367**	**1.167**

*Italic ‐ Inclusion in multivariate analysis*; Bold ‐ significant in multivariate analysis

No other medications, including ACE inhibitors, AT1 receptor antagonists, beta-blockers, statins, antidiabetics, or anticoagulants, showed statistically significant associations in either univariable or multivariable models.

These findings demonstrate that L-thyroxine and class III opioids are independently associated with hepatic steatosis in the studied population.

### MASLD has no influence on cognitive performance in the elderly

Table 4 shows details on cognitive and neuropsychological assessments in the cohort. Cognitive performance, assessed by the Mini Mental State Examination (MMSE), did not differ significantly between patients with and without hepatic steatosis (median 24 vs. 25; p = 0.795). Analysis of individual MMSE components likewise showed no group differences. In addition, neither the clock-drawing test (p = 0.961) nor the geriatric depression scale (p = 0.931) revealed significant differences between groups (Table 4). Overall, these results suggest that hepatic steatosis was not associated with significant alterations in global or domain-specific cognitive performance in this elderly population.

**Table 4:** Cognitive function

	No MASLD (*n*=149) Median (IQR)	MASLD (*n*=147) Median (IQR)	*p*-value
MMSE	25 (19 – 27)	24 (18 – 28)	0.694
1. orientation	9 (6 – 10)	10 (6 – 10)	0.503
2. registration	3 (3 – 3)	3 (3 – 3)	0.486
3. attention and calculation	4 (2 – 5)	4 (2 – 4)	0.493
4. recall	2 (0 – 2)	2 (0 – 3)	0.821
5. language	3 (3 – 3)	3 (3 – 3)	0.165
6. complex task	3 (3 – 3)	3 (3 – 3)	0.916
7. reading	1 (1 – 1)	1 (1 – 1)	0.919
8. writing	1 (1 – 1)	1 (0 – 1)	0.718
9. constructive tasks	0 (0 -1)	0 (0 -1)	0.405
Shulman CDT	3 (3 – 5)	3 (2 – 4)	0.252
GDSY	4 (3 – 7)	4 (2 – 6)	0.414

Data are presented median and interquartile range. P<0.05 were considered significant. Mann-Whitney U test for scaled variables was used

### Hepatic steatosis index is not useful for steatosis prediction in elderly patients with MASLD.

**Fig. 2:**
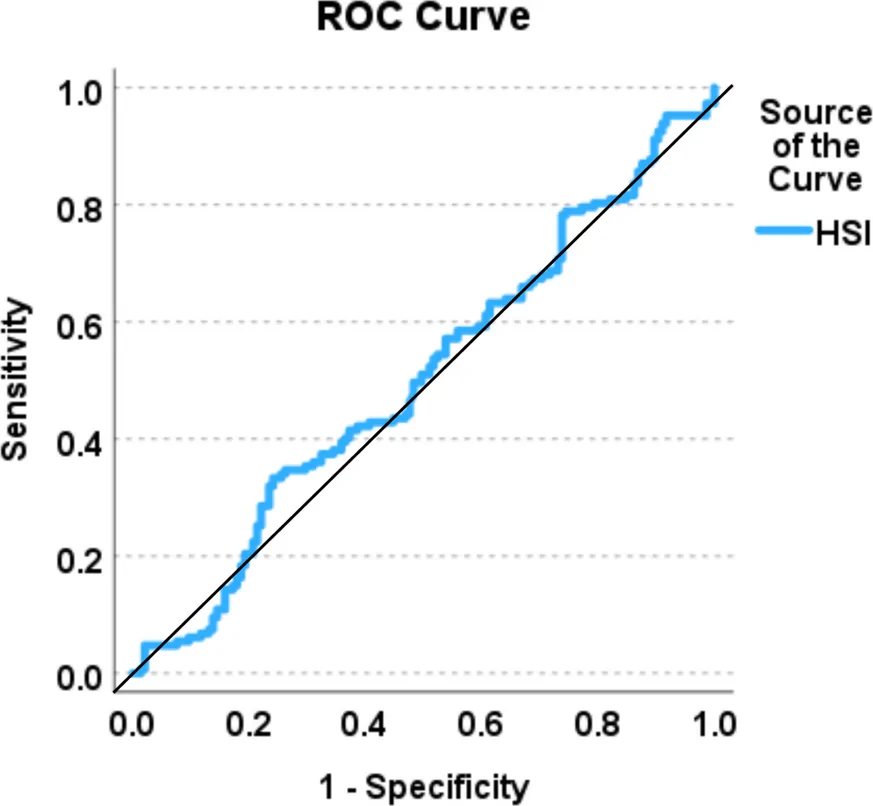
ROC Analysis for HIS. ROC analysis for the hepatic steatosis index (HIS) with ultrasound evidence of hepatic steatosis as reference. The area under the ROC curve is 0.503. Details of the ROC analysis are shown in Supporting Table 1

Next, we analyzed the predictive capacity of the hepatic steatosis index for steatosis prediction in our cohort. We had available data for 292 patients (steatosis: n = 147; no steatosis: n = 145). The area under the ROC curve (AUROC) revealed an area of 0.503, indicating no predictive capacity for HSI in this cohort (Fig. 2). Details on AUROC analysis are summarized in Supp. Table 1. Furthermore, details on test metrics (including percentage of correctly classified patients) at standard cutoffs are shown in Supp. Table 2. This analysis confirms that currently used scores, despite not having age as a variable, are not useful for MASLD prediction in elderly patients.

### Increased hand grip strength is associated with a higher likelihood of hepatic steatosis

**Fig. 3:**
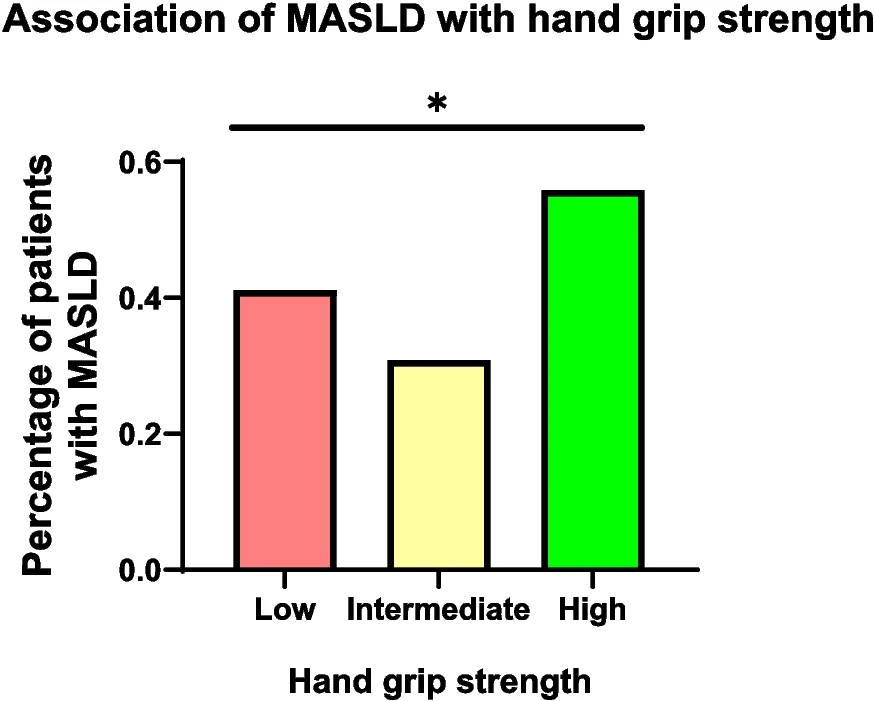
Percentage of MASLD according to hand grip strength. Barplot showing percentage of MASLD in patients stratified by their hand grip strength into three groups according to tertiles (low, intermediate, high). Data are presented as frequencies. P values were calculated using the Kruskal–Wallis test. *P < 0.05

Since sarcopenia in the medical records was identified to be more prevalent among patients with hepatic steatosis compared to patients without steatosis, we analyzed the influence of hand grip strength on the prevalence of hepatic steatosis. For this analysis, patients with available hand grip strength (n = 204) were classified in patients “low”, “intermediate” and high” hand grip strengths according to terciles. Cut off for low hand grip strength was < 11.6 kg, while patients with high hand grip strength demonstrated values > 16.6 kg. Patients with high grip strength showed higher proportions of hepatic steatosis (Fig. 3). In multivariable regression analysis for hand grip strength, including age, sex, BMI, diabetes, hepatic steatosis and depression, only sex was independently associated with hand grip strength (p < 0.001) (Supp. Table 3).

### Hepatic steatosis is not a predictive discriminant of functional status after geriatric hospitalization

Since, we have previously demonstrated that presence of hepatic steatosis is associated with higher handgrip strength and lower prevalence of sarcopenia, we finally analyzed the impact of hepatic steatosis on the functional performance of the patients in our study cohort.

Therefore, an ordinal regression analysis was conducted to identify factors associated with functional autonomy at discharge, categorized as autonomous, semi-autonomous, or dependent (Supporting Table 4). Patients with in-hospital death were excluded. The model included clinical variables such as Tinetti score, Barthel index, presence of hepatic steatosis, diabetes, and sarcopenia.

The model fit was statistically significant (χ^2^ = 32.768, p < 0.001), suggesting that the model was effective in differentiating between the autonomy states at discharge. The Pseudo R-Square values (*Cox and Snell* = *0.175*) revealed modest overall relationship between the predictors and autonomy state. Presence of hepatic steatosis (MASLD), evaluated as a categorical variable, was not significantly associated with autonomy level (Estimate for No MASLD vs. reference = 0.443, p = 0.166). Similarly, neither diabetes nor hand grip strength showed a significant association with the outcome. The only variable significantly associated with higher autonomy at discharge was Barthel index (Estimate = −0.033, p < 0.001), indicating that lower functional scores were predictive of greater independence. In contrast, the Tinetti score did not significantly predict discharge autonomy (Supporting Table 4).

In summary, among the variables examined, only the Barthel index emerged as a statistically significant determinant of discharge autonomy in this geriatric cohort.

## Discussion

In this retrospective study, we found that the presence of metabolic dysfunction-associated steatotic liver disease (MASLD) was not associated with impaired functional autonomy or cognitive impairment in older adults, mostly aged above 80 years, but higher hand grip strength as a surrogate of preserved muscle function. Moreover, our data suggests that commonly used non-invasive tests should not be used in octogenarians upon further validation. While earlier studies referred to the condition as ‘non-alcoholic fatty liver disease’ (NAFLD), we consistently applied the updated nomenclature ‘metabolic dysfunction-associated steatotic liver disease’ (MASLD) throughout this discussion. However, in case earlier studies used NAFLD criteria, this terminology was kept. Data on the prevalence and clinical outcomes of MASLD in individuals older than 80 years are scarce. In one study including 179 patients above 80 years of age, the prevalence of hepatic steatosis showed an age-related decline: while steatosis was present in 44.5% in adults aged 65–70 years, it declined to 31.8% in those over 80 years. [16]. In contrast, larger cohort analyses that grouped patients within the age range of 60–80 years reported a high prevalence of MASLD ranging from 35.1% to 41.9% [2, 17]. However, the latter study was derived from a nationwide cohort, in which included subjects were predominantly below 60 years of age. Moreover, although metabolic features remain significantly associated with MASLD in older adults aged 65–85 years, the strength of the association between the number of metabolic syndrome components and the likelihood of MASLD appears to diminish with advancing age [2]. Taken together, these findings suggest that, while MASLD is highly prevalent in older adults, its epidemiology and clinical determinants in individuals beyond 80 years are insufficiently understood, underscoring the need for dedicated studies in this age group.

Importantly, in the elderly, MASLD might not be the limiting factor for overall outcome but neurological impairment represents a big healthcare burden [18, 19]. Recent studies show conflicting results with regard to the link between MASLD and neurocognitive decline. In a large-scale analysis of over 600,000 individuals aged > 60 years from the Korean National Health Insurance Service database, a high fatty liver index (FLI > 60) was significantly associated with an increased risk of dementia, while a low FLI was associated with a reduced risk [20, 21]. Notably, in this study more than ¾ of analyzed individuals were aged < 70 years. On the other hand, in the Rotterdam Study, no significant association was found between NAFLD (as defined by either FLI or sonographic criteria) and the risk of dementia or mild cognitive impairment (MCI). Intriguingly, a potential protective effect of NAFLD on cognitive outcomes was observed within the first five years of follow-up [22]. Our findings, focusing on patients > 80 years, suggest that the presence of MASLD in the elderly is not associated with cognitive impairment, as recorded through multiple assessments. We used the MMSE and CDT as established cognitive screening tools, whereas the GDS-15 is a depression screening instrument used to account for depressive symptoms as a potential confounder in the assessment of cognitive function. The inclusion of the GDS-15 is therefore of particular value, as depressive symptoms may otherwise lead to misclassification of patients as cognitively impaired. [23]. Of note, earlier trials on MASLD in older adults did not incorporate a depression scale since it is frequently not recorded.

Risk stratification for the development of fibrosis is primarily based on FIB-4 or NFS in primary care setting [24, 25]. All of them incorporate age as a variable prognosticating the risk for fibrosis development. Therefore, it is not recommended to use non-invasive tests for steatosis or fibrosis prediction in patients with an age greater than 65 year [26]. This is why we tested the HSI in our analysis, since age is not included. This assessment is validated in younger populations as a non-invasive test of hepatic steatosis. According to our database, with an AUROC of 0.503, HSI was not useful in this cohort of octogenerians. In these patients, none of currently recommended non-invasive tests are recommended nor validated. However, some tests were proposed to be used irrespective of age, such as APRI and NI2 + ™ for fibrosis prediction [27]. However, it should be noticed that age ≥ 65 years built the oldest group, which again does not match with the age of our cohort. This underscores the importance of the need for specific non-invasive tests suitable for geriatric patients and their respective outcomes.

Another aim of our study was to investigate the relationship between MASLD in older adults and sarcopenia since the literature is conflicting and data in elderly population are scarce. A positive correlation between MASLD and sarcopenia had been established in numerous studies in younger adults aged 40–60 years [28–31]. Moreover, hand grip strength as a surrogate of sarcopenia, was also associated with MASLD in older adults (approx. 52—70 years) in a Korean, nationwide study [32]. Nevertheless, in these cited studies, patients were younger than in our cohort. Our data suggest that, in octogenarians, MASLD may be inversely associated with sarcopenia [33]. We are not aware of studies which specifically investigated the relationship between MASLD and sarcopenia, underscoring the need for future research in this area.

Finally, our findings implicate that MASLD in adults aged > 80 years, might not predict poorer functional outcomes at discharge, including autonomy status. Our findings challenge the prevailing notion that MASLD uniformly contributes to adverse health trajectories [34], at least in octogenerians. This highlights the importance of age-specific investigations into the systemic manifestations of MASLD. Not surprisingly, in our analysis, Barthel index at admission predicted functional outcome, which have been reported previously in geriatrics and palliative care [35]. Further factors, such as sarcopenia itself, hand grip strength or age or BMI were not independently associated with functional outcome after geriatric rehabilitation in our cohort.

This study has several notable strengths including a large, well-characterized cohort of older adults, mainly aged 80 and above, undergoing inpatient geriatric rehabilitation, and therefore allowing for the assessment of clinically relevant outcomes such as functional autonomy, cognitive status, and physical performance. The use of standardized assessments, including hand grip strength and validated tools for cognitive and functional evaluation, enhances the reliability of our findings. On the other hand, our study has limitations inherent to the retrospective design. Selection bias may be present due to the single-center setting and the specific patient population referred for rehabilitation, which may not be generalizable to all older adults with MASLD. Another limitation of our study is the absence of a direct muscle mass assessment (either bioelectrical impedance analysis- or dual-energy X-ray absorptiometry-derived assessments or lumbar muscle cross-sectional area by cross-sectional imaging), which is recommended by the EWGSOP2 consensus for the diagnosis of sarcopenia [36]. A further limitation is that hepatic steatosis was diagnosed using ultrasound rather than by histological confirmation, which may cause misclassification bias. Lastly, residual confounding by unmeasured variables, such as nutritional status, and inflammatory markers, cannot be excluded. Liver fibrosis was not recorded which renders a more granular stratification difficult. Finally, most studies cited in the discussion included younger individuals which makes the comparison of our data with existing literature complicated. However, we performed an unselected screening of patients, where all patients were included in the analysis irrespective of pre-existing conditions. Despite these limitations, the study provides first insights into the complex and, as according to our report, counterintuitive relationships between MASLD, sarcopenia, cognitive function, and functional outcomes in the geriatric population.

In this cohort of elderly patients, MASLD was not associated with cognitive performance or functional recovery after hospitalization. While diabetes mellitus was more common among patients with hepatic steatosis, sarcopenia was unexpectedly more prevalent in those without steatosis. Commonly applied predictive tools such as the hepatic steatosis index lacked discriminatory value in this age group. Interestingly, greater hand grip strength might be linked to a higher likelihood of steatosis, highlighting the complex relationship between muscle function and liver fat in the elderly. Taken together, these findings suggest that MASLD in older adults presents with unique associations distinct from those observed in younger populations, underscoring the need for age-specific diagnostic and management strategies.

## Supplementary Information

Below is the link to the electronic supplementary material.

Supplementary file1 (DOCX 20 KB)

## Data Availability

Data is not available due to GDPR.

## Declarations

### Disclaimer

The funders had no role in study design, data collection and interpretation, or the decision to submit the work for publication.
