# Supplementary material for: MASLD in the oldest-old: lack of association with cognition or functional autonomy and poor predictive utility of the hepatic steatosis index
Source: GeroScience. 2025 Dec 24;48(6):7613–25. doi: 10.1007/s11357-025-02060-3 (PMC13638829; doi:10.1007/s11357-025-02060-3)
Supplement: Supplementary file 1 [file 11357_2025_2060_MOESM1_ESM.docx]

**SUPPORTING TABLES - MASLD in the Oldest-Old: Lack of Association with Cognition or Functional Autonomy and Poor Predictive Utility of the Hepatic Steatosis Index**

**Supporting Table 1 – Hepatic steatosis index (HSI) as predictor for hepatic steatosis. Area under the ROC curve analysis**

| AUROC Analysis | | | | | |
| --- | --- | --- | --- | --- | --- |
| Test result variable | Area | Std. Error | Asymptotic Significance | Asymptotic 95% Confidence Interval | |
|  |  |  |  | Lower Bound | Upper Bound |
| Hepatic Steatosis Index | 0.503 | 0.034 | 0.922 | 0.437 | 0.570 |

**Supporting Table 2 – Test metrics for hepatic steatosis index (HSI) at standard cutoffs**

| HSI Performance at standard cutoffs | | | | |
| --- | --- | --- | --- | --- |
| Test | Cut off | Accuracy (correctly classified) | Sensitivity  [95%-Confidence interval] | Specificity  [95%-Confidence interval] |
| Hepatic Steatosis Index < 30 for ruling out steatosis | < 30 | 51.71 % | 0.2621  [0.1973 to 0.3391] | 0.7755 [0.7015 to 0.8354] |
| Hepatic Steatosis Index > 36 for ruling in steatosis | > 36 | 51.92 % | 0.6759 [0.5960-0.7467] | 0.3605 [0.2874-0.4408] |

**Supporting Table 3 – Multivariable linear regression**

| Multivariable linear regression for hand grip strength | | | | | | |  | |  |
| --- | --- | --- | --- | --- | --- | --- | --- | --- | --- |
|  | Unstandardized |  | Standardized |  |  | 95% Confidence Interval | | | |
|  | B | Std. Error | B | t | Sig. | Lower Bound | | Upper Bound | |
| (Constant) | 1.369 | 1.002 | 0.089 | 1.365 | 0.174 | -0.609 | | 3.346 | |
| Fettleber | -0.147 | 0.089 | -0.109 | -1.644 | 0.102 | -0.323 | | 0.029 | |
| Age | 6.338 | 1.086 | 0.389 | 5.835 | <.001* | 4.195 | | 8.48 | |
| Sex(w-1;m-2) | 0.083 | 0.091 | 0.06 | 0.906 | 0.366 | -0.097 | | 0.263 | |
| BMI_Tx | -0.843 | 1.366 | -0.04 | -0.617 | 0.538 | -3.537 | | 1.851 | |
| Depression | 1.369 | 1.002 | 0.089 | 1.365 | 0.174 | -0.609 | | 3.346 | |
| Hand grip strength | -.026 | .021 | 1.469 | 1 | .225 | -.067 | | .016 | |
| Diabetes | .252 | .313 | .652 | 1 | .420 | -.360 | | .865 | |
| Hepatic Steatosis | .366 | .303 | 1.467 | 1 | .226 | -.226 | | .959 | |

**P<0.05 (*) was considered significant.**

**Supporting Table 4 – Ordinal Regression**

| Ordinal Regression | | | | | | | | | | | |  | |
| --- | --- | --- | --- | --- | --- | --- | --- | --- | --- | --- | --- | --- | --- |
| *Model Fitting Information* | | | | | | | | | | | |  | |
| Model | | -2 Log  Likelihood | | Chi-Square | | df | | Sig. | | | |  | |
| Intercept Only | | 423.436 | |  | |  | |  | | | |  | |
| Final | | 394.237 | | 29.200 | | 7 | | <.001 | | | |  | |
|  | |  | |  | |  | |  | | | |  | |
| *Goodness-of-Fit* | |  | |  | |  | |  | | | |  | |
|  | | Chi-Square | | df | | Sig. | |  | | | |  | |
| Deviance | | 394.237 | | 518 | | 1.000 | |  | | | |  | |
|  | |  | |  | |  | |  | | | |  | |
| *Pseudo R-Square* | |  | |  | |  | |  | | | |  | |
| Cox and Snell | | 0.15 | |  | |  | |  | | | |  | |
|  | |  | |  | |  | |  | | | |  | |
| *Variable Estimates* | | | | | | | | | | | |  | |
|  |  | |  | |  | |  | |  |  | 95% Confidence Interval | | |
|  |  | | Estimate | | Std. Error | | Wald | | df | Sig. | Lower Bound | | Upper Bound |
| Threshold | Autonomous [1] | | .358 | | 2.708 | | .017 | | 1 | .895 | -4.949 | | 5.666 |
|  | Semiautonomous [2] | | 1.574 | | 2.710 | | .337 | | 1 | .561 | -3.738 | | 6.885 |
|  | Independent [3] | | 4.850 | | 2.745 | | 3.121 | | 1 | .077 | -.531 | | 10.231 |
| Variables | BMI | | -.001 | | .026 | | .001 | | 1 | .977 | -.052 | | .050 |
|  | Age | | .043 | | .027 | | 2.490 | | 1 | .115 | -.010 | | .097 |
|  | Barthel | | -.024 | | .009 | | 7.579 | | 1 | .006* | -.040 | | -.007 |
|  | Hand grip strength | | -.026 | | .021 | | 1.469 | | 1 | .225 | -.067 | | .016 |
|  | Diabetes | | .252 | | .313 | | .652 | | 1 | .420 | -.360 | | .865 |
|  | Hepatic Steatosis | | .366 | | .303 | | 1.467 | | 1 | .226 | -.226 | | .959 |

**P<0.05 (*) was considered significant.**
